# Determination of drug-like properties of a novel antileishmanial compound: *In vitro* absorption, distribution, metabolism, and excretion studies

**DOI:** 10.4103/0253-7613.56075

**Published:** 2009-08

**Authors:** Susanta Kumar Mondal, Nirup B. Mondal, Sukdeb Banerjee, Upal Kanti Mazumder

**Affiliations:** Department of Pharmaceutical Technology, Jadavpur University, India; 1 Steroid and Terpenoid Chemistry Division, Indian Institute of Chemical Biology, 4 Raja SC Mullick Road, Kolkata - 700 032, India

**Keywords:** Antileishmanial compound, absorption, distribution, metabolism and excretion, parallel artificial membrane permeability assay, rat liver microsome, stability, solubility

## Abstract

In drug discovery research, the compounds should not only to be potent and selective but also must possess acceptable pharmacokinetic properties such as absorption, distribution, metabolism, and excretion (ADME) to increase success rate in clinical studies.

**Objective:**

Exploration of drug-like properties of 2-(2-methylquinolin-4-ylamino)-N-phenyl acetamide, a potent antileishmanial compound by performing some *in vitro* ADME experiments along with validation of such studies.

**Materials and Methods:**

Experimental protocols were established and validated for stability (in PBS pH7.4, simulated gastric and intestinal fluid), solubility, permeability, distribution coefficient (Log D), plasma protein binding and metabolism by rat liver microsomes by using spectrophotometer or HPLC. Methods were considered valid if the results of the standard compounds matched with reported results or within acceptable range or with proper ranking (high-medium-low).

**Results:**

The compound was found to be stable (>95% remaining) in all stability studies and aqueous solubility was 299.7 ± 6.42 μM. The parallel artificial membrane permeability assay (PAMPA) indicated its medium permeability (Log Pe = −5.53 ± 0.01). The distribution coefficients (Log D) in octanol/PBS and cyclohexane/PBS systems were found to be 0.54 and −1.33, respectively. The plasma protein binding study by the equilibrium dialysis method was observed to be 78.82 ± 0.13% while metabolism by Phase-I enzymes for 1 hour at 37°C revealed that 36.07 ± 4.15% of the compound remained after metabolism.

**Conclusion:**

The methods were found to be very useful for day-to-day ADME studies. All the studies with the antileishmanial compound ascertained that the compound bears optimum pharmacokinetic properties to be used orally as a potential drug for the treatment of leishmaniasis.

## Introduction

It has been estimated that in spite of molecular modeling toward specific molecular target, 30% of compounds fail to show efficacy, while 50% of active compounds fail to become a successful drug due to poor pharmacokinetics or toxicity.[1] Thus, early evaluation of pharmacokinetic (ADME) properties has become mandatory to increase success rate in clinical studies. In recent years, many *in vitro* approaches have been developed for evaluation of such properties to speed up the discovery process, reduce failure rate at the final stage, minimize time and cost, and also to avoid complexities associated with animal experiments. These approaches not only show good correlation with *in vivo* findings but also have accelerated the drug-discovery process to a great extent.[2–4]

Oral delivery is the most convenient and desirable route of drug administration, which demonstrates for absorption-related studies along with distribution, metabolism, and excretion that are common for all types of formulation irrespective of the route of administration. Stability and dissolution of the drug compound in the gastro-intestinal (GIT) fluid; permeation through gastro-intestinal barrier; binding to plasma proteins; distribution throughout the body; metabolism and excretion are some of the crucial parameters to be evaluated for an oral drug, and an active compound should have all these properties to an optimum level to become a successful drug.

**Figure 1:**
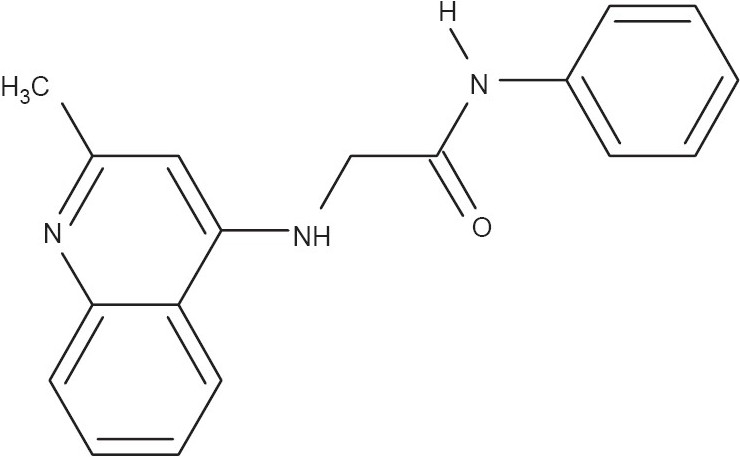
Structure of 2-(2-Methylquinolin-4-ylamino)-N-phenylacetamide

Leishmaniasis is a global health problem especially in tropical and subtropical countries and approximately 350 million people are estimated to be prone to this fatal disease.[5] But there are few drugs developed as of yet, which can counteract the problem efficiently. Moreover, the existing antileishmanial compounds bear toxicity especially to heart and liver.[6] In continuation of our research for lead identification of antileishmanial agents, several indolylquinoline derivatives were synthesized[7] and evaluated, both *in vitro* and *in vivo,* against promastigote and amastigote forms of parasite of *Leishmania donovani*[8–10] The compound, 2-(2′′-dichloroacetamidobenzyl)-3-(3′-indo yl)-quinoline, having significant antileishmanial activity was further undertaken for structure activity relationship.[7–10] As a result, a number of analogs were prepared and the compound, 2-(2-methylquinolin-4-ylamino)-*N*-phenylacetamide (henceforth termed as S-4, Figure 1) was found to be significantly more effective than sodium antimony gluconate in reducing the parasite burden both in spleen and liver.[11,12]

The present study investigates the drug-like properties of S-4 in regards to stability, solubility, absorption, distribution, plasma protein binding (PPB), and metabolism, contemplating that the compound will be formulated as an oral drug. Also the experimental protocols were validated to check their usefulness in providing reliable data.

## Materials and Methods

### Materials

Solvents for HPLC were procured from Qualigens Fine Chemicals (Mumbai, India). Nicotinamide adenine dinucleotide phosphate (NADP), glucose-6-phosphate, glucose-6-phosphate dehydrogenase, magnesium chloride, and trifluoroacetic acid (TFA) were purchased from Sisco Research Laboratories Pvt. Ltd. (Mumbai, India). Dodecane was obtained from Fluka Chemie Gmbh (Switzerland). All other chemicals and standard drugs were purchased from Sigma, USA. Human plasma was obtained from Institute of Blood Transfusion Medicine and Immuno-Haematology, Kolkata, India. The test compound 2-(2-methylquinolin-4-ylamino)-*N*-phenylacetamide (S-4) was prepared as described.[11,12]

### High performance liquid chromatography

Waters HPLC (Model: Alliance 2996) with PDA detector (Model 2477) and XTerra RP18 column (250 × 4.6 mm, 5 μ) were used for the present study. The ratio of water and acetonitrile (ACN), both containing 0.1% TFA, was varied for different standard drugs following the standard protocol with a total flow rate of 1 ml/min and run time not more than 15 minutes. The system volume was 2.50 ± 0.02 ml. For S-4, the mobile phase was 30:70 of water:Acetonitrile (both containing 0.1% TFA) with a run time of 10 minutes. The retention time for S-4 was found to be 6.7 minutes. Validation of HPLC methods was based on specificity, reproducibility (%RSD for RT and area < 2% with 10 injections of same sample), and linearity.

### Spectrophotometry

The absorption maxima (λ_max_) for the test compound and the standard drugs were determined using Spectramax Gemini (Molecular Devices, USA). The absorbance-based studies were performed at λ_max_ of each compound, and for S-4, λ_max_ was detected at 240 nm.

### Validation studies

Some standard drugs with reported results were studied along with S-4 for the validation of each of the experimental methods (described below) except chemical stability studies. The experimental methods were considered valid if the results of the standard compounds matched with reported results or within acceptable range or with proper ranking (high-medium-low).

### Chemical stability

A 100-μM solution of S-4 was prepared in phosphate buffered saline (PBS), pH 7.4, from a stock of 25 mM in DMSO and was kept at 25°C for 24 hours. Percentage stability was determined based on chromatographic peak area of the sample kept for 24 hours and that of 0 hour (freshly prepared and injected) sample. All the samples were prepared in triplicate.

Similar studies were carried out at 37°C replacing PBS with simulated gastric fluid (SGF) and simulated intestinal fluid (SIF) which were prepared following United States Pharmacopoeia, and stability was checked for upto 2 hours for SGF and 4 hours for SIF.

### Solubility

A 4 μl of 25 mM stock (DMSO) of S-4 was diluted to 200 μl with PBS (pH 7.4) and incubated at 25°C for 2 hours with constant shaking. The undissolved compound, if any, was filtered through the PVDF membrane filter (Millipore, MA). To 120 μl of the filtrate, 80 μl of acetonitrile was added and mixed thoroughly. The concentration of S-4 in the filtrate was determined spectrophotometrically from linearity (5-400μM) of the compound prepared in PBS containing 40% acetonitrile. The observed solubility result was multiplied by 200/120 to account for the acetonitrile addition in the test filtrate. Similar studies were also performed for five standard drugs (diclofenac sodium, caffeine, haloperidol, diethylstilbesterol, and tamoxifen) for solubility ranking. The results beyond the linearity range were reported as solubility >400 μM or <5 μM, as applicable.

### Parallel artificial membrane permeability assay

Millipore acceptor and donor plate (Cat No: MAPB310 and MASSACCEPTOR) were used in the study. The artificial membrane was prepared by adding 5 μl of 1% lecithin in dodecane to the donor wells. 150 μl of 250 μM test compound in PBS pH 7.4 (5% DMSO) was added to donor wells and the donor plate was placed onto the acceptor plate (containing 300 μl PBS pH 7.4, 5% DMSO) ensuring proper contact between the membrane and acceptor liquid without entrapment of an air bubble. Incubation was carried out for 16 hours at room temperature in a water-saturated chamber to avoid evaporation from donor wells. Such experimental conditions were chosen to check validity and acceptability of the method as reported earlier.[13] After incubation, 250-μl acceptor samples were taken out and absorbance measured at λ_max_ of each compound. To a 200 μl acceptor sample, a 100 μl donor solution was added, mixed properly, and this mixture was termed as “equilibrium.” The absorbance of the equilibrium was also determined.

Five standard compounds of different permeability (high: Propranolol HCl, carbamazepine; medium: Warfarin; low: Furosemide, methotrexate) were also studied to make the permeability ranking. The integrity of the artificial membrane was checked by the Lucifer yellow permeation study as reported earlier.[13]

(1) $$\text{Calculation: Log Pe}=\text{Log}[\text{Cx-}\ln\text{ (1}-{\text{OD}}_{\text{Acceptor}}/{\text{OD}}_{\text{Equilibritum}})]$$

where C = (V_D_ × V_A_)/[(V_D_ + V_A_)× Area (cm^2^) × porosity × time (seconds)]

Where V_D_ = volume of donor (ml), V_A_ = volume of acceptor (ml).

A linear graph was plotted with observed and reported values for the standard drugs. The experimental method was considered valid for permeability ranking if linear correlation coefficient (R^2^) was more than 0.95.

### Distribution coefficient (Log D) in octanol/PBS pH 7.4

Octanol and PBS pH 7.4 with ratio 1:1 (v/v) were taken in a flask and shaken mechanically for 24 hours to pre-saturate PBS with octanol and octanol with PBS. These pre-saturated solvents were used for the present study.[14,15] A 4 μl of 25 mM compound in 400 μl PBS was allowed to undergo partitioning with different volumes of octanol (100, 150, 200, and 400 μl). After phase mixing by vigorous shaking for 2 hours, phase separation was done by centrifugation at 3000 rpm for 5 minutes followed by 1 hour standing without disturbance. The PBS layer was taken out. A 100 μl acetonitrile was added to a 100 μl PBS aliquot and absorbance was measured along with the reference samples (400 μl PBS containing 4 μl of 25 mM compound + 400 μl acetonitrile). Five standard compounds were similarly studied to check the validity of the method.

Log D (the logarithm of concentration ratio of compound in organic and aqueous phase) was calculated according to Dellis *et al.:*[15]

(2) $$\text{Log D}=\text{Log}[\{({\text{OD}}_{\text{Reference}}-{\text{OD}}_{\text{Sample}})\times {\text{V}}_{\text{PBS}}\}/{\text{OD}}_{\text{Sample}}\times {\text{V}}_{\text{Octanol}})]$$

where V_PBS_ = volume of PBS and V_Octanol_ = volume of octanol.

Log D for each volume ratio was determined and each Log D should be within average Log D ± 0.3 log units and for standard compounds average Log D should fall within ± 0.3 log unit of reported results along with the above criteria in force.[15]

### Distribution coefficient Log D in cyclohexane/PBS pH 7.4

Log D in cyclohexane/PBS was performed following the similar method as done for Log D in octanol/PBS. Here, 5 mM compound stock was used to prevent precipitation problem with this system and volumes of cyclohexane were 150, 200, 400, and 800 μl. Five standard compounds were similarly studied to check the validity of the method where acceptance criteria remained same as in Log D octanol/PBS pH 7.4.

### Plasma protein binding

The binding of compound to human plasma was determined using equilibrium dialysis with 6-hour incubation with constant shaking at 37°C as per the method described by Kaplita and Liu.[4] The initial concentration of the compound used was 10 μM (DMSO ≤1%). Quantitations were done by HPLC. Standard drugs (ranitidine HCl, caffeine, methotrexate, propranolol HCl and warfarin) of different %PPB were also studied to check whether %PPB values of the standard compounds match with the reported values.

### Metabolism by liver microsomal enzymes

Ethical clearance was obtained from Animal Ethics Committee of Indian Institute of Chemical Biology for use of animals for the present study. Rat liver microsome was prepared in house following the method by Li[16] and protein estimation was done as described by Lowry *et al.*[17] The reaction mixture was prepared in 100 mM phosphate buffer pH 7.4 where 10 μM test compound was allowed to undergo metabolism by 0.5 mg liver microsomal protein/ml for 1 hour at 37°C in the presence of NADPH regeneration system (3.3 mM glucose-6-phosphate, 3.3 mM MgCl_2_, 1.3 mM NADP and 0.4 U/ml glucose-6-phosphate dehydrogenase) and 1 mM EDTA.[18,19] The reaction was quenched by adding equal volume of ice-cold acetonitrile followed by a centrifugation at 14,000 rpm for 15 min.[20] The supernatant was injected to HPLC for quantitation. The % stability (% compound remaining after metabolism) was calculated as follows:

(3) % Compound remaining=100×(peak area of 1 hour sampl/peak area of 0 hour sample)

For 0 hour sample, the compound was added after addition of ice-cold acetonitrile and then vortexed, followed by centrifugation as mentioned above.

Standard drugs atenolol, propranolol HCl, imipramine HCl, and verapamil HCl were used as quality control samples. Results within 15% variation from the published values were accepted. The 15% variation was arbitrarily selected because much variation in results was reported with different batches of microsome and also for species variation, inspite of the similar experimental protocol.[18–20]

## Results

It was observed that S-4 was stable (>95%) in PBS pH 7.4, SGF, and SIF. Stability was 99.16 ± 1.32, 101.19± 0.76, and 97.70 ± 0.15%, respectively in the tested conditions. For all other experiments, results of the standard drugs were in accordance with the reported values.[13,19–28] This signified the acceptability of the methods adopted here.

**Figure 2:**
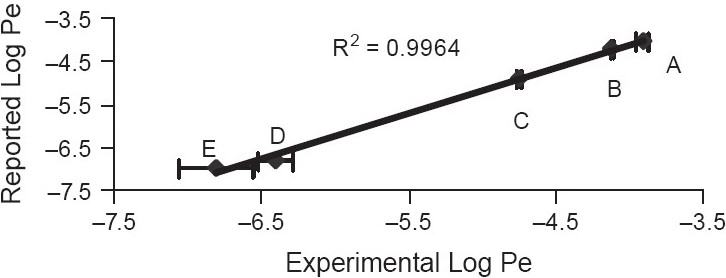
Parallel artificial membrane permeability assay (A) Propranolol HCl; (B) Carbamazepine; (C) Warfarin; (D) Furosemide; (E) Methotrexate. Log Pe of S4 = −5.53 ± 0.01. Results are average of three experiments (n = 7) ± SEM

Solubility of S-4 was 299.70 μM in PBS pH 7.4 [Table 1]. Permeability results are summarized in Figure 2 where compounds of different permeability (high: Propranolol HCl, carbamazepine, medium: Warfarin, low: Furosemide, very low: Methotrexate) were compared with the reported results and S-4 showed medium permeability. It showed Log D values of 0.54 and −1.33 in the octanol /PBS system and in the cyclohexane/PBS system, respectively [Table 2]. Binding to the human plasma protein was 78.82% [Table 3]. Metabolism of different compounds is summarized in Table 4, and S-4 showed 36.07% compound remaining after metabolism.

**Table 1:** Solubility of S-4 and standard drugs in Phosphate buffered slain, pH 7.4

*Compound*	*Solubility (μM)^a^*	*Reported value (μM)*
Diclofenac Sodium	> 400	> 400
Caffeine	>400	> 400
Haloperidol	48.49 ± 3.21	40-63
Diethylstilbesterol	18.94 ± 1.00	5-20
Tamoxifen	6.14 ± 1.63	3-30
S-4	299.70 ± 6.42	-

a Results are average of three experiments ± SEM S-4: 2-(2-methylquinolin-4-ylamino)-N-phenylacetamide

**Table 2:** Log D of S-4 and standard drugs

*Log D octanol/PBS*			*Log D cyclohexane/PBS*		
	
*Compound*	*Result^a^*	*Reported value*	*Compound*	*Result^a^*	*Reported value*
Verapamil HCl	2.31	2.42	Caffeine	−1.28	−1.23
Propranolol HCl	1.17	1.23	Warfarin	−1.28	−1.31
Chloramphenicol	1.13	1.14	Haloperidol	0.52	0.46
Metoprolol tartrate	−0.25	−0.30	Verapamil HCl	1.20	1.12
Furosemide	−1.11	−1.28	Ketoconazole	1.38	1.41
S-4	0.54	−	S-4	−1.33	-

a Results are average of three experiments and in all cases individual Log D values were within ± 0.3 log unit of Average Log D S-4: 2-(2-methylquinolin-4-ylamino)-N-phenylacetamide

**Table 3:** Percentage of plasma protein binding of S-4 and standard drugs

*Compound*	*Result^a^*	*Reported value*
Ranitidine HCl	14.84 ± 1.21	10-19
Caffeine	40.66 ± 0.31	29-43
Methotrexate	50.70 ± 0.36	35-57
Propranolol HCl	80.12 ± 0.61	80-93
Warfarin	98.21 ± 0.24	99
S-4	78.82 ± 0.13	-

a Results are average of three experiments ± SEM S-4: 2-(2-methylquinolin-4-ylamino)-N-phenylacetamide

**Table 4:** S-4 and standard drugs metabolism by rat liver microsomes

*Compound*	*% Parent compound remaining*	
	
	*Result^a^*	*Reported (approx)*
Atenolol	90.33 ± 1.30	100
Propranolol HCl	75.11 ± 1.11	70
Imipramine HCl	42.86 ± 1.19	38
Verapamil HCl	10.31 ± 6.68	5
S-4	36.07 ± 4.15	-

a Results are average of three experiments ± SEM S-4: 2-(2-methylquinolin-4-ylamino)-N-phenylacetamide

## Discussion

Whenever there is a need of high throughput analysis or screening of some properties it is important to have confidence on the reliability of the methods adopted so that no misleading conclusion is made. Therefore, one is required to run quality control samples to check reliability of the methods. It was observed that all the methods used in the present study furnished similar results or within acceptable range as have been reported earlier for different standards[13,19–28] and thus these data as well as the procedures can be utilized for day-to-day analysis of *in vitro* ADME properties.

Stability study in PBS, pH 7.4, and SIF revealed that S-4 is stable at both conditions indicating its acceptability for intended absorption from the high surface area of the intestine. However, its stability in SGF indicated that no special formulation is required to provide protection against the low pH of stomach.

Determination of compound solubility in phosphate buffer saline or water has become an essential early measurement in the drug discovery research. Poor aqueous solubility can cause problems in many different *in vitro* testing techniques, resulting in unreliable results and reproducibility problems. Even larger problem may arise when insoluble precipitates cause false positives in bioassays, potentially wasting time, and significant cost.[21] The standard way to determine the solubility of a compound is to use the shake-flask solubility method.[22] However, this method is labor intensive, renders low throughput, and needs compound in solid form that may be incompatible with how compounds are generally maintained e.g., in DMSO.[23] The solubility of S-4 was found to be 299.70 μM in PBS, pH 7.4 [Table 1]. This indicated that the compound is not poorly soluble and may not create solubility-related problems when formulated as oral dosage form.

Bioavailability of drugs is influenced by various factors like stability in gastrointestinal fluid, solubility etc. Permeability of a compound through the gastrointestinal tract is one of the most important aspects in deciding whether the molecule is a potential lead candidate or not. Cell-based assays using Caco-2 or MDCK cell lines are commonly used.[24,29,30] But the technique is time consuming, costly, and labor intensive, while non-cell-based assays are automation compatible, relatively fast, inexpensive, and straight forward, and also the data arising from such assays correlate well with human drug absorption values.[24] However, cell-based studies are required to measure active transport as well as to know if the compound is a potential P-glycoprotein (P-gp) substrate or not.[24,29] The permeability study by PAMPA aims to determine absorption potential of a compound through passive diffusion. It is well known that more than 80% of orally administered drugs enter the blood stream by passive diffusion through intestinal epithelium. In the present study, 1% lecithin in dodecane was used as the artificial membrane. However, use of different types of lipids is also reported.[27,28] The results of the present study correlate well with the reported values (R^2^ = 0.9964, [Figure 2]) and S-4 was found to possess medium permeability.

Log D value of S-4 was determined to be 0.54 and −1.33 in Octanol/PBS and cyclohexane/ PBS systems, respectively [Table 2]. Log D (Octanol/PBS) determines lipophilicity of compounds and thus determines the ability/inability of the drug to cross the gastrointestinal barrier.[3] High Log D (>3) leads to low solubility, erratic/poor absorption, high plasma binding, whereas very low Log D (<0) indicates low permeability and high renal clearance. Compounds with moderate Log D (0-3) have good balance between solubility and permeability and are optimal for oral absorption and cell membrane permeation in cell-based assays.[3,14,15] S-4 (Log D = 0.54) falls in the moderate Log D group and, therefore, bears moderate permeability as well as solubility as observed here [Table 1 and Figure 2]. On the other hand, negative Log D (Log D = −1.33) of S-4 in cyclohexane/PBS is indicative of its inability to cross blood–brain barrier. Penetration through blood-brain barrier is not required for treatment of leishmaniasis, rather inaccessibility to brain may be beneficial in avoiding brain-related toxicities.

Plasma protein binding (PPB) is another important parameter that needs to be evaluated in the ADME domain, as unbound drug plasma concentration decides the pharmacological effects of a compound in regards to tissue distribution, cell entry, receptor interaction, and availability for elimination.[21,26] Previous reports suggest that dialysis for 6 hours is optimum to reach equilibrium[26] and hence 6-hour dialysis was performed. S-4 showed 78.82% binding to plasma proteins that is quite acceptable and close to that of propranolol HCl [Table 3].

For orally formulated dosage forms, the drug, upon absorption, reaches the liver where it undergoes metabolism by liver microsomal enzymes and becomes inactive. Thus metabolic stability of a compound plays an important role in bioavailability and overall pharmacokinetic performance. Typically, liver microsomes from different species (human/rat/dog/monkey) are used to screen the extent of metabolism by the Phase-I metabolizing enzymes.[19] Rapid screening of microsomal stability at an early stage of the drug discovery process provides information on the potential metabolism-related liabilities of the compounds and provides an early alert to the medicinal/synthetic chemists to modify the compound. However, a variety of reports are available with varying assay conditions (LM: 0.1-1 mg/ml, test substance: 0.5-15 μM, DMSO: 0.2-1%, ACN: 0.2-2%) resulting in a general lack of standard assay conditions as well as high variability among different laboratories.[16,18–20] In the present study, the methodologies reported by Di *et al.,*[19] for the optimization of a higher throughput microsomal stability screening assay for profiling drug discovery candidates, was utilized. Assay conditions were optimized so that the results matched with the report (optimization data not shown here). DMSO concentration was also kept below 0.2% to avoid inhibition of Phase-I enzymes by the solvent. HPLC was chosen as analytical tool for the detection of parent compound remaining. LC-MS/MS is generally used for stability studies where initial compound concentration is 1 μM or less. But in the present metabolic stability study, the compound concentration was 10 μM. HPLC can provide satisfactory data at this concentration and thus the use of costly instruments like LC-MS/MS was avoided. We have already standardized and reported the suitability of conventional HPLC in microsomal stability assay.[31] The present study showed 36.07% of S-4 remaining after metabolism [Table 4], which indicates acceptable metabolic stability as well as indication of hepatic clearance.

It is also noteworthy that toxicity studies, performed in our laboratory, with S-4 revealed no alarming sign to different organs tested.[11]

## Conclusion

Some articles are available to determine physicochemical properties of a compound. But, so far the literature is concerned each paper deals with one type of property and such procedures may or may not be automation possible. In the present study, very simple and also automation possible methods for estimation of some physicochemical parameters have been described. Verifying the observed results with reported values, the acceptability of each of the methods was checked. In all cases, the results were consistent and in accordance with the standard reports. The compound S-4 showed excellent stability in PBS pH 7.4, SGF, and SIF; good solubility, and medium permeability, whereas Log D values indicated its good pharmacokinetic nature. PPB and LM stability studies also supported the drug-like pharmacokinetic property. All the studies ascertain that S-4 bears optimum pharmacokinetic properties and can be formulated for oral delivery.
